# A single day fasting may increase emergency room visits due to renal colic

**DOI:** 10.1038/s41598-021-86254-7

**Published:** 2021-03-22

**Authors:** Dorit E. Zilberman, Tomer Drori, Asaf Shvero, Yoram Mor, Harry Z. Winkler, Nir Kleinmann

**Affiliations:** Department of Urology, Chaim Sheba Medical Center, affiliated to Sackler School of Medicine, Tel-Aviv University, Tel-Hashomer, Ramat-Gan, 52621 Tel-Aviv, Israel

**Keywords:** Urology, Epidemiology, Renal calculi

## Abstract

We aimed to explore whether a single-day of fasting (SDF) increase emergency room (ER) visits due to renal colic (RC). We elected to concentrate on Yom-Kippur (i.e.: SDF), the holiest day in Judaism. Food and liquid consumption is prohibited during this day for 25 h, and an estimated 50–70% fasting rate is observed. SDF always takes place between mid-September and mid-October during which the temperature in the Middle-East ranges between 19 and 30 °C. ER visits for RC between 01/2012 and 11/2019 were reviewed, and the Gregorian days on which SDF occurred were retrieved. The number of ER visits for RC was compared between SDF and the surrounding days/months as well as to another single-day "standard" holiday (SDSH) that precedes SDF in 10 days and is not associated with fasting. Of 11,717 ER visits for RC, 8775 (74.9%) were males. Male:Female ratio was 3:1. The mean daily number of ER visits for RC during the 3 days following SDF was 6.66 ± 2.49, significantly higher compared with the mean annual daily visits (4.1 ± 2.27, *p* < 0.001), the mean daily visits during the week prior to SDF (5.27 ± 2.656, *p* = 0.032), and the mean daily visits during September (5.06 ± 2.659, *p* = 0.005), and October (4.78 ± 2.23, *p* < 0.001). The mean number of ER daily visits for RC during the 3 days following SDSH, 5.79 ± 2.84, did not differ compared with the mean daily visits during September and October (*p* = 0.207; *p* = 0.13, respectively). It was lower compared to SDF, however statistically insignificant (*p* = 0.285). A single-day fasting may increase ER visits for RC. The mechanism underlying this phenomenon is unknown.

## Introduction

Short-term intermittent fasting has become a popular lifestyle approach for weight loss and health improvement^1–3^. It is also common practice during the Muslim month of Ramadan as well as on the Jewish holiest of days, Yom Kippur (the Day of Atonement). This observance is not very likely to negatively affect a healthy individual, however, there may possibly be adverse effects among individuals with a history of nephrolithiasis.

Several studies have explored the effect of observing the fast during Ramadan on the number of emergency room (ER) admissions due to renal colic (RC)^4–8^.

They reported no adverse effects of intermittent fasting on RC events, however, they either studied small samples or conducted the analyses during a single calendar year^5,6^. Moreover, the Muslim calendar is based on a lunar year, whereupon the month of Ramadan may occur in a different season every few years.

Notably, fasting continues from sunrise to sunset, and the absolute length of the daily fast changes in accordance with the season, varying between 6 and 18 h^5^. All but one study^7^ noted this important feature, and it was also the only one to consider that fasting during Ramadan may increase the number of ER visits due to RC.

We explored the effect of fasting on the number of RC events from the vantage of Jewish religious practices. We designed this study to determine whether the day of Yom Kippur that dictates a 25-h-long uninterrupted fast increases ER visits due to RC events.

## Patients and methods

### Data collection

Following approval from our Institutional Review Board (# SMC-18-5352), we retrospectively reviewed all ER visits due to RC between January 2012 to November 2019. Those visits were identified using ER discharge records with a diagnosis of “RENAL COLIC” or 788.0 (renal colic, International Classification of Disease, 9th revision, Clinical Modification ICD9-CM code). These codes were assigned by an ER physician or urologist to patients with flank pain or other symptoms suspicious for renal colic (i.e.: abdominal pain, lower urinary tract symptoms), and with sonographic or computerized tomographic imaging studies that had demonstrated obstructing urinary tract calculi. The dates of the visits as well as the patients' age and sex were also recorded.

### Study population

Our institution is one of the largest tertiary university-affiliated medical centers in the Middle East region. Geographically, it is located in the mid-portion of the coastal plain.

The vast majority of the population which lives in this region is Jewish.

Due to ethical and political restraints, the patients' ethnicity and religion are not coded in the system.

The Jewish population served by the emergency department is at least 18 years of age, and it includes ultra-orthodox, conservative, and liberal Jewish men and women.

### The single day fasting

Yom Kippur is a single-day fasting (SDF) during which Jews are commanded to fast.

The exact rate of fasting during this 25-h period has never been reported, however, public opinion polls have estimated an overall fasting rate between 50 and 70%, with the highest rate among the ultra-orthodox Jews (97–100%) and the lowest rate among the liberal Jews (35–40%).

The SDF (i.e., abstinence from all foods and liquids) starts at sunset and lasts uninterruptedly for 25 h.

It always takes place in the same season of the year, and always falls between mid-September and mid-October.

During this time of the year the temperature in the coastal plain ranges between 19 and 30 °C (66.2–86 °F).

### Exemptions

Oral medications are not permitted during fasting hours. Dispensation from strict observance is clear-cut which exempt a person from fasting where it may adversely affect physical or mental health. People with chronic illnesses can seek medical advice to determine whether they may be exempted from fasting. The elderly, pregnant women, and breastfeeding mothers are also exempt.

### Data analysis

The annual, monthly and daily numbers of ER visits due to RC were calculated. Patients' age and sex have been analyzed as well.

The Gregorian days on which SDF occurred were extrapolated and listed (Table 1). ER visits due to RC on SDF were compared to the daily visits for the same etiology 7 days before and a 7 days after SDF.

**Table 1:** Gregorian days of Yom Kippur fasting (i.e.: single day fasting, SDF) between 2012 and 2019.

Gregorian year	SDF
2012	September 25th–26th
2013	September 13th–14th
2014	October 3rd–4th
2015	September 22nd–23rd
2016	October 11th–12th
2017	September 29th–30th
2018	September 18th–19th
2019	October 8th–9th

ER visits due to RC during SDF were also compared to the mean annual daily ER visits due to RC and to the mean daily ER visits for RC during September–October period.

Each patient’s age and sex were included in this analysis.

Another comparison has been made between SDF and another single-day "standard" holiday at the same season (SDSH) that precedes SDF in 10 days and is not associated with fasting (i.e.: The Hebrew New Year Day).

The statistical analysis was performed with the Statistical Package for Social Sciences (SPSS, Version 25.0, Chicago, IL, USA).

Data are given as mean/standard deviation (SD) unless otherwise specified. Kolmogorov–Smirnov test was used to assess the normality of data distribution. For normal distribution continuous variables student's t-test was applied.

For non-normal distribution continuous variables Mann–Whitney test was applied. Binomial test has been used to compare proportions (male / female).

A *p* value of less than 0.05 was considered statistically significant.

### Ethics approval

The present study was performed in accordance with the declaration of Helsinki and was approved by Chaim Sheba Medical Center ethics committee, approval # SMC-18-5352. Given the retrospective nature of this study, informed consent has been waived by the above mentioned committee.

## Results

Between 2012 and 2019, there were 11,717 ER visits due to RC, among which 8775 (74.9%) were by males and 2942 were by females with a mean age of 46.9 ± 14.4 years.

The mean daily number of ER visits for RC was 4.1 ± 2.27, with a male:female ratio of 3:1.

**Figure 1:**
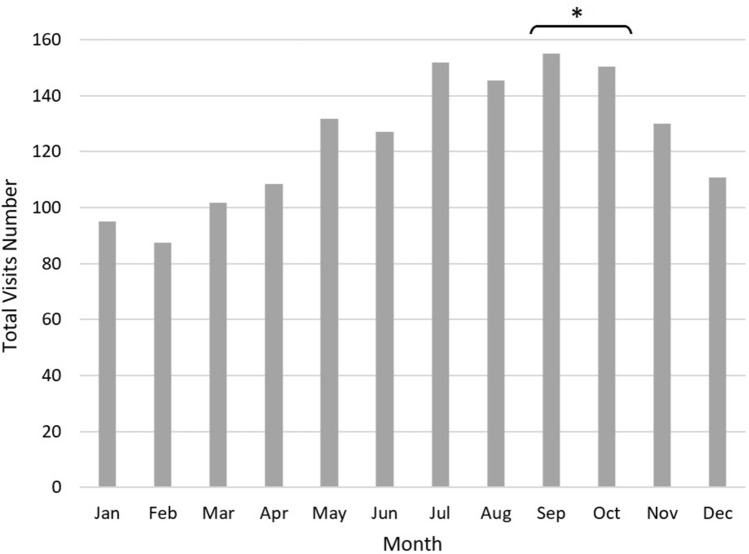
Average monthly emergency room visits due to renal colic. *Months in which Yom Kippur (single day fasting) occurred.

The highest monthly mean number of ER visits for RC was recorded during July–August–September–October (151.8 ± 13.5, 145.3 ± 13.3, 155.1 ± 12.1, and 150.3 ± 12, respectively) (Fig. 1).

**Figure 2:**
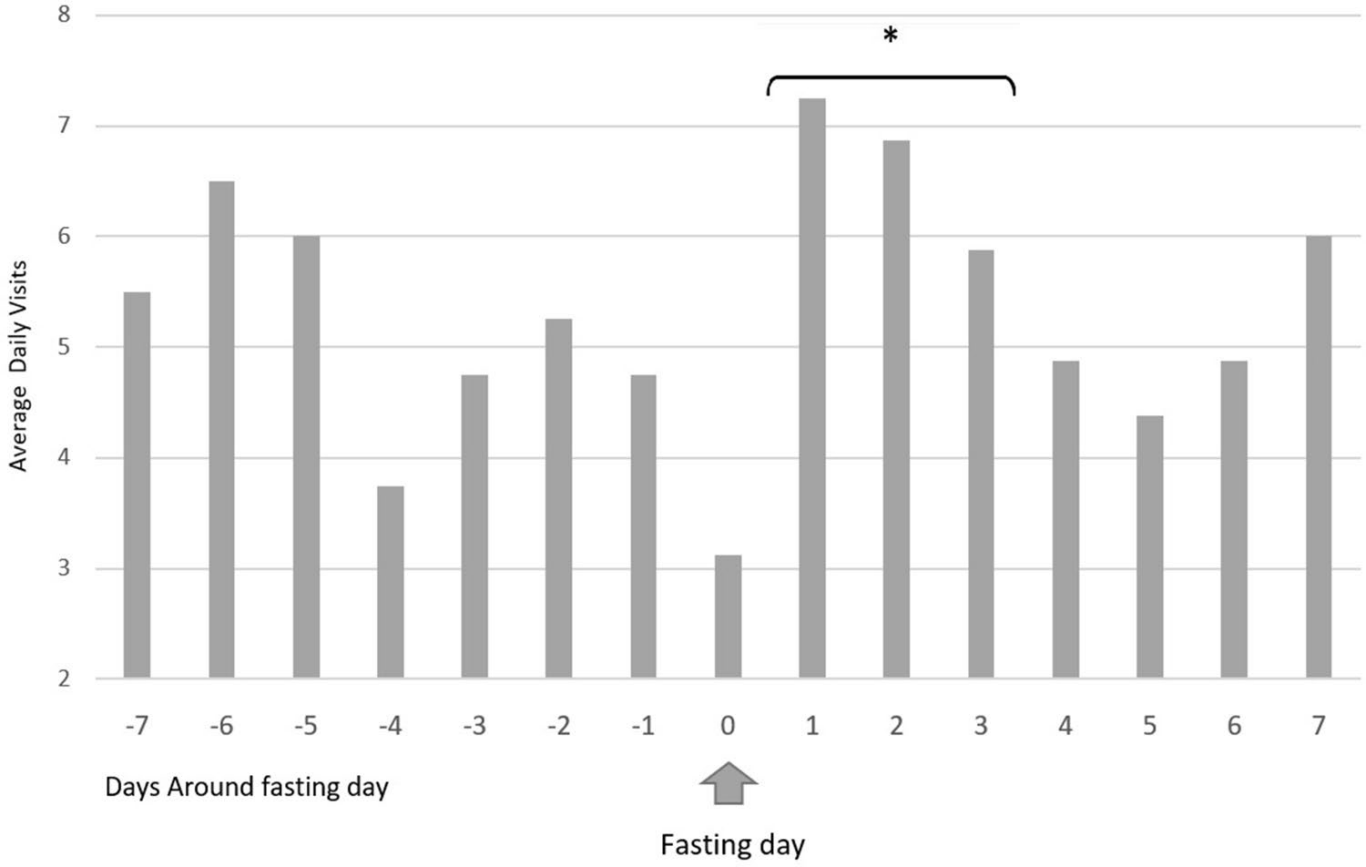
Average daily emergency room visit distribution around the day of Yom Kippur (single day fasting). *Average daily visits during the first 3 days following Yom Kippur.

The mean daily number of ER visits for RC during the 3 days following SDF was 6.66 ± 2.49 (Fig. 2, Table 2). This rate was significantly higher compared with the mean annual daily visits (*p* < 0.001 Confidence Interval (CI) 1.57–3.31), compared with the mean daily visits during the week prior to SDF (5.27 ± 2.656, *p* = 0.032, CI 0.12–2.665), and compared with the mean daily visits during September (5.06 ± 2.659, *p* = 0.005, CI 0.49–2.72), and those during October (4.78 ± 2.23, *p* < 0.001, CI 0.8–2.97).

**Table 2:** Number of ER referrals due to RC around Yom Kippur (i.e.: single day fasting—SDF).

Gregorian year	Days relative to SDF															
	− 7	− 6	− 5	− 4		− 3	− 2	− 1	0	**1**	**2**	**3**	4	5	6	7
2012	9	9	4	2		7	8	3	1	**8**	**4**	**12**	3	7	4	4
2013	3	10	7	3		1	4	2	5	**9**	**11**	**5**	6	4	7	4
2014	3	2	5	8		5	4	5	4	**9**	**7**	**7**	4	2	6	6
2015	6	8	8	5		5	9	8	1	**3**	**6**	**2**	4	4	2	9
2016	2	4	5	2		5	3	3	5	**8**	**9**	**5**	4	4	9	6
2017	11	8	6	5		4	7	7	2	**6**	**7**	**5**	5	6	6	4
2018	6	5	10	0		1	6	7	0	**8**	**7**	**8**	10	2	1	5
2019	4	6	3	5		10	1	3	7	**7**	**4**	**3**	3	6	4	10
Average	5.5	6.5	6	3.75		4.75	5.25	4.75	3.12	**7.25**	**6.87**	**5.87**	4.87	4.37	4.87	6

The 3 days following SDF are bolded.

The male:female ratio was 4.03:1 for the ER visits during the 3 days following SDF, which was higher compared with the general male:female ratio, but did not reach statistical significance (*p* = 0.097).

The mean age of the patients presenting to the ER for RC during the 3 days following SDF was not significantly different from the total mean age.

The mean daily number of ER visits for RC during the 3 days following SDSH was 5.79 ± 2.84. Though lower compared with the 3 days following SDF, it did not reach statistical significance (*p* = 0.285). Yet, in oppose to the 3 days following SDF, this value was not statistically significantly higher compared with the mean usual daily visits during September (*p* = 0.207) and October (*p* = 0.130).

## Discussion

The precise physiologic mechanisms that adversely affect the formation of nephrolithiasis during prolonged fasting remain uncertain.

One study observed that the Ramadan fast had caused a significant decrease in both systolic and diastolic blood pressures and a resultant decrease in renal perfusion and urine flow rate in normotensive males^9^.

Another study^10^ focused on the risk factors for stone formation during the Ramadan period in a population of 57 young healthy male candidates, 37 of whom were previous stone formers. Comparison of urine samples between the two groups before fasting and during 15 h of fasting revealed an overall total decrease in urine volume and urine calcium concentration as well as a decrease in total excretion of calcium, phosphor and magnesium and a total increase in concentrations of uric acid, citrate, phosphor sodium, and potassium during fasting.

Urinary calcium-phosphate super-saturation during fasting was significantly low, while uric acid super-saturation was significantly high.

No change in urinary calcium-oxalate super-saturation was observed during fasting. Those authors concluded that there was no evidence that the Ramadan fast significantly increased the risk of stone formation.

Shafiee et al.^11^ focused on CaHPO_4_ precipitates since they serve as the main precursors of calcium-oxalate monohydrate nucleation and growth.

Their study group was comprised of 15 healthy individuals who had observed 18 h of fasting and whose laboratory findings showed a significant decrease in mean urine flow, but no increase in calculated risk of CaHPO_4_ precipitation.

Unlike the small populations in these earlier studies, our study sample consisted of thousands of individuals whose ages ranged from 18 years to the very elderly and who represented both sexes. In addition, the findings were gathered throughout 8 consecutive years.

The impact of hot and dry weather on renal colic events and kidney stone disease has been extensively investigated. A recent review article^12^ focused on 13 studies that analyzed seasonal/monthly variations in ER presentations with RC.

In all but one study, the higher the outside temperature, the higher was the expected incidence of kidney stone events worldwide.

It has been assumed that dehydration combined with inadequate fluid intake and a resultant low urine volume may contribute to kidney stone formation^13^.

One study demonstrated a proportional increase in urinary calcium excretion with outdoor temperature increase as well as increased urinary calcium-oxalate and calcium-phosphate supersaturation^14^.

Another study^15^ demonstrated a time lag of ≤ 3 days between an outdoor temperature rise and the rise in patient presentations to ER with stone formations.

This very small time gap may be indicative that the stones are formed at a relatively rapid rate.

These findings are in line with the current ones that the highest numbers of ER visits due to RC were recorded during the warmest months of the year—July through October. Taken together with the effects of fasting, we demonstrated an almost twofold increase in the average ER visits for RC 3 days following SDF.

In our study, the overall male:female ratio was 3:1, similar to the one reported elsewhere^12^ but lower than the 5:1 rate reported in a study also coming from the Middle East^16^. Interestingly, we demonstrated that a single day of fasting increased this rate to 4.03:1, with male patients generally being at a higher risk for a stone event compared to female patients, and especially when warm weather and prolonged fasting are combined.

A recent study^17^ suggested a protective mechanism of estrogen against stone formation. They reported that incubating renal tubular cells with 17β-estradiol for 7 days resulted in changes in cellular proteome of the renal tubular cells and a consequent decrease in surface expression of calcium-oxalate crystal receptors, decreased intracellular metabolism, and enhanced cell proliferation and tissue healing, altogether assumed to contribute to stone formation prevention.

Our findings did not support their hypothesis, since we had included peri- and post-menopausal women and their inclusion should have decreased the male:female ratio during the study period.

We contend that other yet undefined mechanisms are involved in the relatively high male:female ratio seen following SDF.

The question of whether or not to fast when one has a known history of nephrolithiasis can be answered by the findings of the present study.

Given the warm weather in the Middle East at the months surrounding SDF and SDSH (average high temperature of 31 °C/87°F in September and 28 °C/83°F in October), it is possible that the high outdoor temperature combined with prolonged fasting may increase the likelihood of an ER visit for RC. If a single day of fasting is a matter of personal choice, individuals with nephrolithiasis should rethink their decision and at the very least preserve fluid intake.

There are limitations in this study that bear mention.

The first is its retrospective nature and the resultant inability to confirm that each individual in this study actually fasted during SDF, obliging us to rely on the assumption that the overall fasting rate among the Jewish population during SDF is around 70%. Interestingly, the present results support this assumption.

The second limitation is the lack of electronic documentation on co-morbidities in the study cohort that also could affect renal conditions.

Last but not least, holidays in general may serve as confounders in the present study. Although the average daily visits following SDF has been shown to be statistically significantly higher compared to the general average ER daily visits for RC, it still remained unclear whether a non-fasting holiday during the hot season would have the same effect. We managed to demonstrate a smaller average daily visits during the three days following SDSH compared to SDF, however given the relatively small observations number (i.e.: total of 21 daily measurements of ER referrals for each holiday), we failed to prove statistical significance. Yet, this number was quite similar to the mean ER daily visits for RC during September and October in oppose to the mean visits number during the three days following SDF, that was significantly higher. We do believe that higher number of observations will better establish our null hypothesis.

In summary, there is an increase in the average number of ER visits due to renal colic following a single-day abstinence of food and liquid intake. Males are more prone to a renal-related event following such stringent fasting compared to females. The precise mechanism(s) underlying this phenomenon has yet to be determined.

## Data Availability

The datasets used and/or analysed during the current study are available from the corresponding author on reasonable request.

## Abbreviations

ER: Emergency room
RC: Renal colic
SDF: Single-day fasting
SDSH: Single-day "standard" holiday
CI: Confidence interval
